# First experimental assessment of *Phlebotomus mascittii* vector competence for *Leishmania infantum* and *Leishmania martiniquensis*

**DOI:** 10.1186/s13071-025-07228-6

**Published:** 2026-01-21

**Authors:** Jovana Sadlova, Anna Hoskova, Katharina Platzgummer, Edwin Kniha, Tomas Becvar, Petr Volf, Vit Dvorak

**Affiliations:** 1 Department of Parasitology, Faculty of Science, Charles University, Prague, Czech Republic; 2 Institute of Specific Prophylaxis and Tropical Medicine, Center for Pathophysiology, Infectiology and Immunology, Medical University of Vienna, Vienna, Austria

**Keywords:** *Phlebotomus mascittii*, *Leishmania infantum*, *Leishmania martiniquensis*, Vector competence, Experimental infection, Europe, Epidemiology

## Abstract

**Background:**

*Phlebotomus mascittii* is one of the most widespread but least studied sand fly species in Europe, occurring from Mediterranean to Central European regions. Despite its broad distribution, its potential role in *Leishmania* transmission remains unknown, mainly due to the lack of laboratory colonies. This study provides the first experimental assessment of the vector competence of *P. mascittii* for *Leishmania infantum* and *Leishmania martiniquensis*.

**Methods:**

Wild-caught *P. mascittii* females from Styria, Austria, were experimentally infected using membrane feeding with blood containing *L. infantum* and *L. martiniquensis* isolates of different geographical origins. Infections were evaluated 7 days post-blood meal (PBM) by microscopy and polymerase chain reaction (PCR). Morphological forms of *L. infantum* were quantified and compared with infections in *Phlebotomus perniciosus*, a known competent vector.

**Results:**

Fifteen (94% of dissected) *P. mascittii* females developed *L. infantum* infections, all showing colonization of the stomodeal valve, whereas *L. martiniquensis* failed to establish infection. Infection patterns and parasite localization closely resembled those in *P. perniciosus*. Morphometric analysis revealed a significantly higher proportion of metacyclic and leptomonad forms and fewer nectomonads in *P. mascittii* than in *P. perniciosus*.

**Conclusions:**

Our findings demonstrate that *P. mascittii* supports full development of *L. infantum* to the transmissible metacyclic stage and colonization of the stomodeal valve, confirming its potential vector competence. This study provides the first experimental evidence on *P. mascittii* vectorial competence and highlights its epidemiological relevance in Europe.

**Graphical Abstract:**

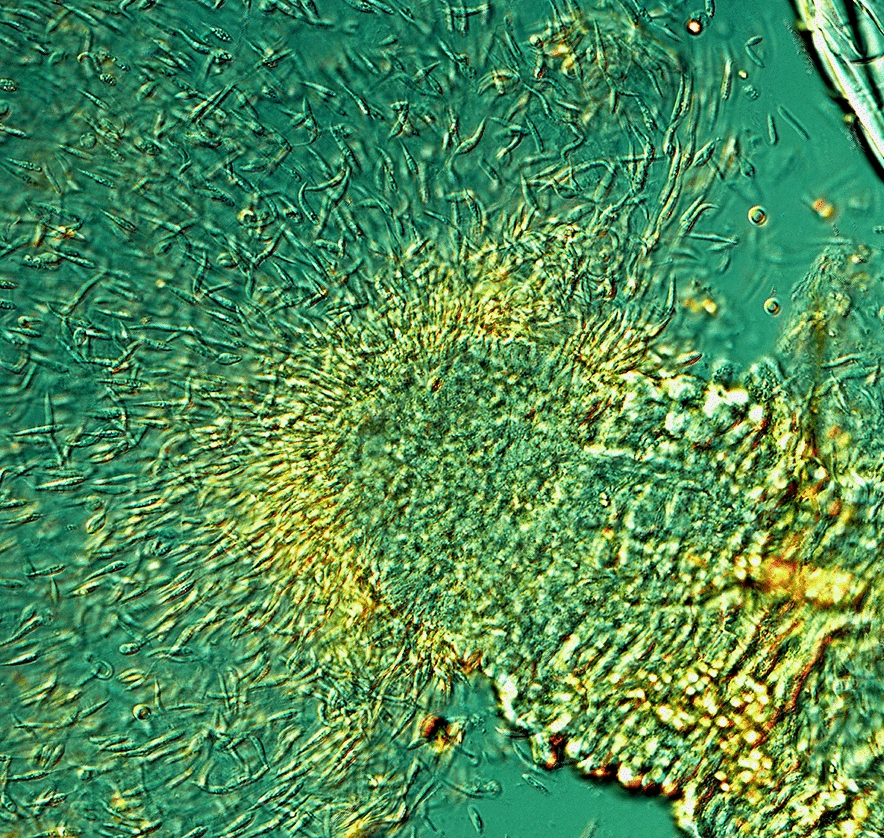

## Background

Leishmaniases are vector-borne diseases of humans and animals caused by protozoan parasites of the genus *Leishmania* (Kinetoplastida: Trypanosomatidae), transmitted mainly by the bite of infected sand flies (Diptera: Psychodidae). Endemic in 99 countries and responsible for an estimated 700,000 to 1 million new cases annually, leishmaniases remain important yet neglected diseases. Their incidence is increasing in several regions due to political instability, human migration, and ongoing climatic and environmental changes [1]. Europe is among the endemic regions, with persistent transmission in traditional foci and increasing risk of introduction of novel *Leishmania* species [2].

Among the European sand fly fauna, *P. mascittii* is one of the most enigmatic species, exhibiting a remarkably wide geographical range. It occurs in most highly endemic Mediterranean countries [3], except for the eastern Mediterranean, where historical records are now attributed to other recently described *Transphlebotomus* species [4, 5]. Notably, *P. mascittii* has a wide northern distribution, with stable or sporadic populations documented in Luxembourg, Germany, Switzerland, Austria, Slovakia, and Hungary [6, 7]. Outside Europe, its occurrence has been reported only once, in northern Algeria [8], indicating that the species is largely confined to the European sand fly fauna. A recent modeling study suggests post-glacial expansion from a single refugium, with further northward spread expected under ongoing climate change [3].

Given its broad and expanding distribution beyond classical sand fly-endemic areas, understanding the potential role of *P. mascittii* in pathogen transmission is of growing importance. In the established epidemiological framework of leishmaniases in Europe, various permissive *Larroussius* species are proven or suspected vectors of *L. infantum*, the main etiological agent widely distributed across vast regions of the Mediterranean basin. Meanwhile, *Phlebotomus sergenti* is recognized as the specific vector of *Leishmania tropica* in small foci in Greece [9]. Recent experimental studies, however, have challenged traditional vector associations, showing that several *Larroussius* species can also support the development of *Leishmania tropica* [10, 11], *Leishmania major*, and *Leishmania donovani* [12]. The role of *P. mascittii* in this context has remained unclear.

Moreover, recent findings suggest that *L. martiniquensis* (subgenus *Mundinia*), a potentially underdiagnosed pathogen of veterinary relevance, also circulates in Europe. *Leishmania martiniquensis* has been previously identified in geographically distant areas such as Martinique, Florida (USA), Brazil, and Thailand, where it has been shown to cause both visceral leishmaniasis (VL) and cutaneous leishmaniasis (CL) in humans as well [13]. Moreover, since 2010, sporadic cases of skin lesions caused by *L. martiniquensis* have been repeatedly reported in cattle and horses in Germany, Switzerland, Austria, and the Czech Republic [14–16]. In most of these countries, *P. mascittii* and *P. perniciosus* are the most common or solely present sand fly species, making them prime candidates for evaluating their vector competence for this *Leishmania* species.

In this changing epidemiological landscape, understanding the vector competence of *P. mascittii*—a widespread European species—remains a key question. However, progress in assessing this has been impeded by the unavailability of a laboratory colony [17]. Our repeated attempts to establish the *P. mascittii* colony remained unsuccessful, as the females refused to feed on multiple blood sources, becoming autogenous and therefore unsuitable for experimental membrane feeding.

In the present study, we used wild-caught *P. mascittii* females from monospecific sites in Styria, Austria, regularly monitored and confirmed to be *Leishmania*-free [18], to assess the susceptibility and development of *L. infantum* and *L. martiniquensis*. These experiments provide the first laboratory data addressing the potential vector competence of *P. mascittii*. *Phlebotomus perniciosus* was used as a control species, as it is a well-established permissive vector and the main vector of *L. infantum* and shares part of its range with *L. martiniquensis* cases.

## Methods

### Sand flies and *Leishmania*

Sand flies were collected at three previously described localities in Austria, namely Hummersdorf, Laafeld, and Unterpurkla [17]. Centers for Disease Control and Prevention (CDC) miniature light traps equipped with fine mesh collection bags (John W. Hock Co., Gainesville, FL, USA) were deployed twice during the sand fly season, each time for two consecutive nights, in July and August 2025. Traps were set at dusk and retrieved before dawn to maximize survival of captured specimens. Live sand flies were aspirated from collection bags, transferred into cages, and maintained under standard conditions during transport as described previously [19].

A laboratory colony of *P. perniciosus* (originating from Spain), a proven vector of *L. infantum*, was used as a reference for infection parameters. *Leishmania infantum* (MCAN/IT/2003/ARIS, Italy) and *L. martiniquensis* (MHOM/TH/2019/Cu2, Thailand; MEQU/CZ/2019/Aig1, Czechia) were cultured in M199 medium (Sigma-Aldrich) supplemented with 10% heat-inactivated fetal calf serum (Gibco), 2% sterile urine, 1% Basal Medium Eagle vitamins (Sigma-Aldrich), and 250 μg/ml amikacin (Medochemie Ltd.).

### Experimental infections of sand flies

Promastigotes from logarithmic-phase cultures were resuspended in heat-inactivated, defibrinated ram blood at a concentration of 1 × 10^6^ promastigotes/ml. Due to the limited number of *P. mascittii* females, both *Leishmania* species were pooled in the same feeder, and the species identity of promastigotes in fed females was confirmed molecularly (see below). Females were allowed to feed through a chick-skin membrane for 2 h. Fully engorged females were maintained under the same standard laboratory conditions as the colony and dissected on day 7 post-blood meal (PBM); corresponding generally to late-stage infection in competent vectors when the blood has already been digested and *Leishmania* parasites colonize the stomodeal valve [20]. Infection intensity and localization were evaluated microscopically and categorized as light (< 100 parasites/gut), moderate (100–1000 parasites/gut), or heavy (> 1000 parasites/gut) following established criteria [21].

To confirm the *Leishmania* species present in infected sand fly guts, a single-step polymerase chain reaction (PCR) targeting the 434-base-pair region of the *Leishmania* *HSP70* gene was performed [22]. PCR products were purified using the Exo-CIP Rapid PCR Cleanup Kit and sequenced with the reverse primer. The resulting sequences were identified by BLAST (Basic Local Alignment Search Tool) comparison with GenBank database entries.

### Morphological analysis of parasites

Parasite morphology from mature infections was examined from methanol-fixed, Giemsa-stained gut smears prepared on day 7 PBM. Promastigotes were observed under oil immersion and photographed using an Olympus DP70 camera.

The body length, body width, and flagellum length of 200 promastigotes per sand fly species were measured using ImageJ software. Promastigotes were classified as follows [23]:

- Metacyclic forms: flagellum length ≥ 2 × body length
- Leptomonad forms: flagellum length < 2 × body length and body length < 14 µm
- Nectomonad forms: flagellum length < 2 × body length and body length ≥ 14 µm

Differences in proportions of morphological forms between sand fly species were analyzed using the Pearson Chi-square test (SPSS software version 27; IBM Corp., Armonk, NY, USA).

### Screening of wild-caught specimens for *Leishmania*

To confirm that trapping localities were free of natural *Leishmania* infections, dead females and unfed females were pooled (five females per pool). DNA was extracted using the High Pure PCR Template Preparation Kit (Roche Diagnostics, Indianapolis, IN, USA) following the manufacturer’s instructions. Pools were screened by PCR targeting of the internal transcribed spacer 1 (ITS1) region of ribosomal DNA using primers LITSR/L5.8S as described previously [24]. DNA extracted from a pool of five *P. mascittii* males from the same locality served as a negative control, while DNA from *P. perniciosus* females infected with *L. infantum* was used as a positive control.

## Results

### Sand fly collection and survival

A total of 168 *P. mascittii* specimens (98 alive, 70 dead) were collected at the three study sites between 15 and 17 July 2025, and an additional 44 specimens (27 alive, 17 dead) between 12 and 14 August 2025. Two experimental infections were performed on the second day after transport to the insectary at Charles University. In total, 63 females were available for the first assay and seven females for the second. None of the females collected dead or females that refused to feed during experiments tested positive for *Leishmania* in PCR screening.

### Experimental infections

**Fig. 1:**
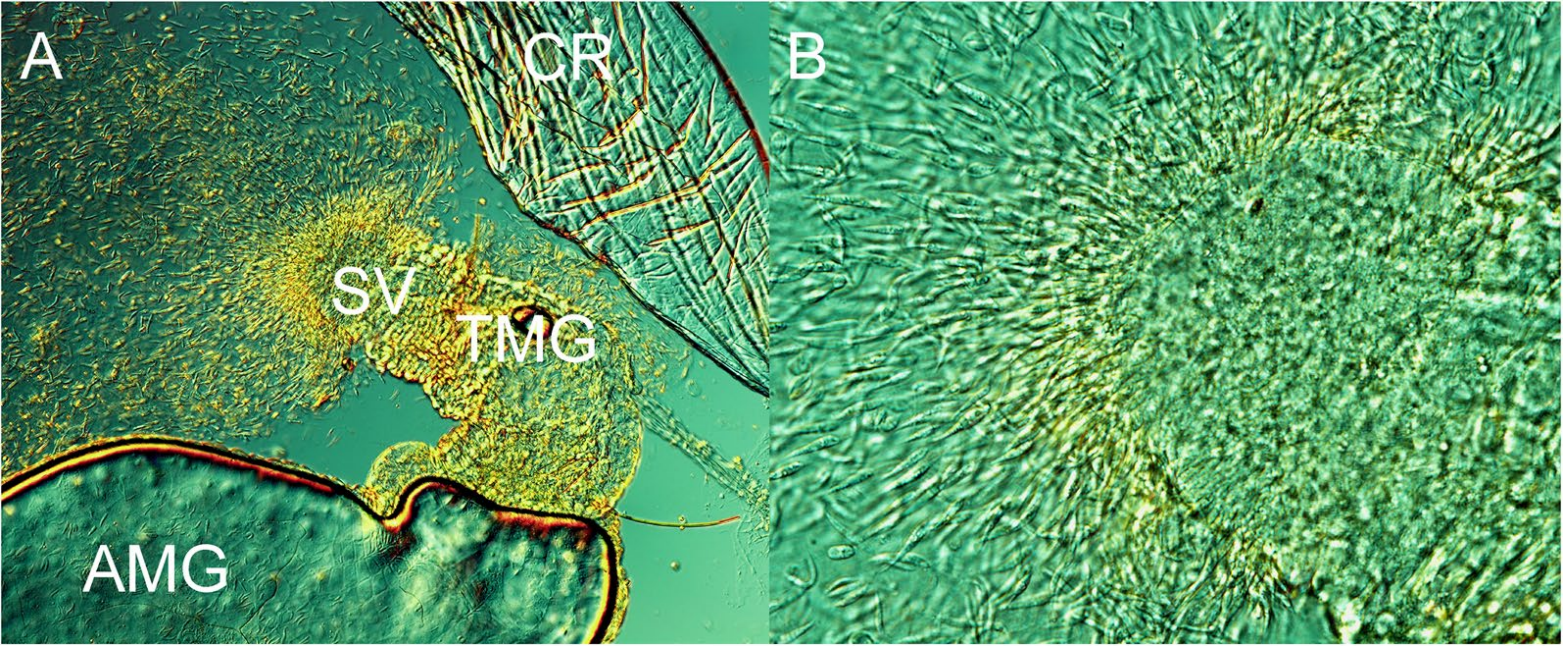
Localization of *L. infantum* infection in *P. mascittii* 7 days PBM. **A** Promastigotes filling the thoracic midgut of *P. mascittii* (×200 magnification). AMG, abdominal midgut; TMG, thoracic midgut; CR, crop; SV, stomodeal valve. **B** Detail of the colonized stomodeal valve (×400 magnification)

In the first experiment, *P. mascittii* females were exposed to *L. infantum* and the Thai isolate *L. martiniquensis* Cu2. Of the 63 females, 29 successfully engorged, and 15 survived until day 7 PBM and were subsequently dissected. In the second experiment, *L. infantum* and the Czech isolate *L. martiniquensis* Aig were used; only two of seven females fed through the membrane, and just one survived until day 7 PBM. Microscopic examination revealed heavy infections in 11 females, moderate infections in four females, and one female without infection. Parasites colonized the stomodeal valve in all 15 infected females (Fig. 1). Molecular analysis confirmed that all infections were caused by *L. infantum*; *L. martiniquensis* did not establish infection in any of the females.

Comparable results were obtained with *P. perniciosus*, a proven vector of *L. infantum*: the parasite established mature infections including colonization of the stomodeal valve, whereas *L. martiniquensis* did not survive to day 7 PBM in any female.

### Morphological analysis

**Fig. 2:**
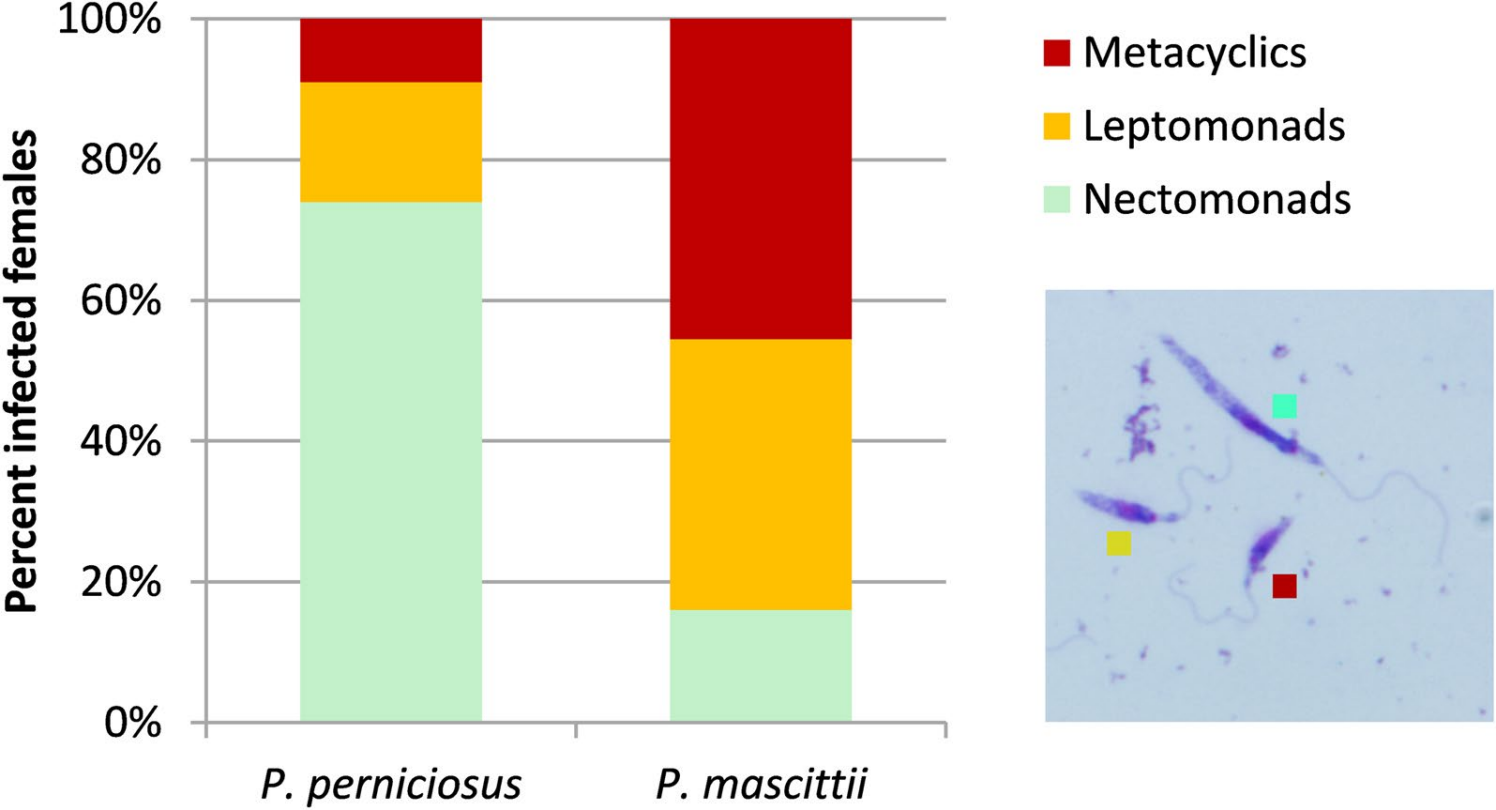
Morphological forms of *L. infantum* in *P. perniciosus* and *P. mascittii* 7 days PBM

The representation of morphological forms of *L. infantum* was analyzed from gut smears on day 7 PBM. Nectomonad, leptomonad, and metacyclic forms were identified in both sand fly species (Fig. 2). In *P. mascittii*, a significantly higher proportion of metacyclic and leptomonad forms and a lower proportion of nectomonads were observed compared to *P. perniciosus* (Pearson Chi-square test: *χ*^2^ = 140.303, *df* = 2, *P* < 0.0001).

## Discussion

This study provides the first laboratory evidence demonstrating the susceptibility of the widespread sand fly species *P. mascittii* to *Leishmania* parasites causing human disease. This represents an important step towards confirming its vector competence. In general, incrimination of a sand fly species as a competent vector requires several criteria to be met: (1) the sand fly feeds on humans and, in zoonotic diseases, on reservoir hosts; (2) it supports the complete development of the parasite after digestion and excretion of the infective blood meal; (3) parasites isolated from wild-caught sand flies are indistinguishable from those from patients; and (4) the sand fly can transmit the parasite by bite [25].

*Phlebotomus mascittii* fulfills the first criterion of anthropophagy—during field captures and attempts to colonize this species, we repeatedly observed the willingness of females to feed on humans. Its generalist feeding behavior further supports its vector potential, as it has been documented to feed on both birds and mammals [18]. The second criterion is supported by molecular evidence, as *L. infantum*-positive *P. mascittii* have been detected by PCR in the Italian island of Montecristo [26] and in Austria [27] and Slovenia [28]. In addition, several cases of leishmaniasis in humans and animals that are assumed to be autochthonous have been described in regions where *P. mascittii* is the only sand fly species observed [18, 26]. However, due to the difficulties in colonizing this species, the remaining two criteria of vector competence have not yet been experimentally verified.

Our experiments with wild-caught *P. mascittii* females infected via a chicken membrane feeding system confirmed that *L. infantum* can survive blood meal defecation and establish heavy mature infections in over 90% of females. Two key prerequisites for transmission—colonization of the stomodeal valve by haptomonad forms and the presence of metacyclic promastigotes—were both met. Colonization of the stomodeal valve, located at the foregut–midgut junction, interferes with subsequent blood-feeding and induces regurgitation of parasites into the host’s skin [29]. Metacyclic promastigotes, the mammal-infective forms characterized by elongated flagella and vigorous motility, were first described decades ago [30] and more recently characterized molecularly as early (dividing) and late (non-dividing) forms [31]. The percentage of metacyclic forms was significantly higher in *P. mascittii* (45%) than in the control species *P. perniciosus* (9%), the dominant vector of *L. infantum* in the Mediterranean region. These findings suggest that *P. mascittii* have the potential to serve as a vector for *L. infantum*. However, it should be considered that non-metacyclic forms are also introduced into the host and may even predominate in the inoculum, as was observed in BALB/c mice bitten by *L. infantum*- and *Leishmania mexicana*-infected *Lutzomyia longipalpis* [32]. Furthermore, it has recently been demonstrated that, along with metacyclics, haptomonads are also transmitted and contribute to exacerbated pathology in mice [31].

In contrast, *L. martiniquensis* failed to establish mature infections in *P. mascittii*. This is consistent with observations in other European sand fly species, namely *P. perniciosus* and *Phlebotomus tobbi*, where the parasite also failed to develop [12]. Similarly, in the Asian vector *Phlebotomus argentipes*, only one of four *L. martiniquensis* strains produced heavy infections, and only in a small proportion of females [33]. In contrast, *L. martiniquensis* developed well in the American biting midge *Culicoides sonorensis*, which successfully transmitted the parasite to mammalian hosts by bite [33]. Together with reports of naturally infected biting midges in Thailand [34–36], these data strongly support the hypothesis that biting midges (Diptera: Ceratopogonidae), rather than sand flies, act as vectors for *L. martiniquensis* and other *Mundinia* species.

Due to the limited number of field-collected females, they were allowed to feed on a pool containing both *Leishmania* species. Promastigotes from mature infections were subsequently identified using molecular methods. Theoretically, competition between these two *Leishmania* species could occur within the sand fly gut. However, co-infections involving two *Leishmania* species performed using the same methodology have not demonstrated such an effect. Chajbulinova et al. [37] reported that *Leishmania turanica* and *L. major* can develop concurrently in *Phlebotomus papatasi*, without any visible sign of competition. Similarly, *L. infantum* and *Leishmania braziliensis* successfully completed their life cycles and produced infective forms in two vector species, *Lutzomyia longipalpis* and *Lutzomyia migonei* [38].

Compared with experimental infections using laboratory-reared colonies, our work was limited by lower numbers of available wild-caught females. Consequently, transmission experiments and tests with additional *Leishmania* species could not be conducted. Future studies should aim to confirm transmission by bite and further assess the vector competence of *P. mascittii*. Nevertheless, the present results provide strong experimental evidence that *P. mascittii* should be regarded as a potential vector of *L. infantum* in Europe.

## Conclusions

This study provides the first experimental evidence that *P. mascittii*, a widespread European sand fly species, is susceptible to infection with *L. infantum* and can support its full development. The key prerequisites for transmission—colonization of the stomodeal valve and presence of metacyclic promastigotes—were successfully met, highlighting the potential vector role of *P. mascittii* in Europe and raising a question of potential vector competence of other species within the subgenus *Transphlebotomus*. In contrast, *L. martiniquensis* failed to establish infections, supporting the notion that other vectors, such as biting midges (Diptera: Ceratopogonidae), may be responsible for its transmission. These findings enhance our understanding of the epidemiology of leishmaniasis in Europe and provide a foundation for future studies on vector capacity of *P. mascittii*.

## Data Availability

All the data are presented in the main manuscript.
